# Supplementary material for: Protein complex prediction for large protein protein interaction networks with the Core&Peel method
Source: BMC Bioinformatics. 2016 Nov 8;17(Suppl 12):37–58. doi: 10.1186/s12859-016-1191-6 (PMC5123419; doi:10.1186/s12859-016-1191-6)
Supplement: Supplementary file 1 — Detailed Experimental settings. Protein complex prediction for large protein protein interaction networks with the Core&Peel Method- Supplementary Materials. Description of the parameters and settings used in testing the competing sw for PC detection in Large PPIN. Description of data sets features distributions. Analysis of Asymptotic Complexity for Core&Peel. (PDF 1284 kb) [file 12859_2016_1191_MOESM1_ESM.pdf]

RESEARCH

Protein complex prediction for large protein protein interaction networks with the Core&Peel Method
Supplementary Materials

Marco Pellegrini*†, Miriam Baglioni and Filippo Geraci

*Correspondence: m.pellegrini@iit.cnr.it
Laboratory for Integrative Systems Medicine - Istituto di Informatica e Telematica and Istituto di Fisiologia Clinica del CNR, via Moruzzi 1, 56124 Pisa, Italy
Full list of author information is available at the end of the article
†Corresponding author

1 Algorithms used for comparisons: brief description

1.1 MCL

MCL (Markov Clustering Process) is an algorithm introduced in [1] for clustering graphs. It simulates in parallel simultaneous random walks from any vertex of the input graph G. The intuition is that for densely connected subgraphs (with sparse connection to the complement in the graph) a random walk is more likely to stay within the densely connected subgraphs within which the walk started. This algorithm has found many applications in bioinformatics (see references in [1]), and it is rather robust and scalable. Thus, it represents a good reference benchmark which often proved to be very competitive against other (early) state of the art methods [2].

1.2 MCL-CAw

MCL-CAw [3] is an algorithm that is based on a refinement of the output of MCL. Inspired by a core-attachment model for protein complexes it finds a more densely connected core within each MCL-detected subgraph. Several experiments show an improved output quality with respect to the pure MCL results for small PPIN.

1.3 COACH

COACH [4] is an algorithm whose main strategy is a greedy local refinement of the *core graph* of a graph $G = (V, E)$. Formally a *core graph* $CG(G)$ is defined as follows. Take any vertex $v \in V$ and a vertex in its 1-neighborhood $u \in N_1(v)$. If the degree of u in the induced subgraph $G[N_1[v]]$ is larger then the average degree in $G[N_1[v]]$, then u is labeled as a *core vertex*. The core graph $CG(G)$ is the subgraph induced in G by the collection of all the *core* vertices of G. Even if the name is similar, the notion of a *core* vertex in ([4]) is quite different form the notion of core number/core decomposition of [5] which is based instead on the *minimum degree* of an induced subgraph.

1.4 MCODE

MCODE [6] is one of the earliest algorithms explicitly designed to cluster PPI networks. Interestingly, it uses the concept of core number for a vertex v, but it computes it not for the whole graph G but locally in the 1-neighborhood subgraph

induced by the vertex v. Our approach involves computing the core number for the whole graph and using these numbers to define suitable 1-neighborhoods. Apparently, changing the order of the two operations has a deep impact on the final quality.

1.5 CMC

The CMC algorithm (Clustering Based on Maximal Cliques) [7] starts by listing all maximal cliques in a graph G by using the algorithm in [8]. Then it performs a merging phase based on the degree of clusters overlap. Our method differs from CMC in several key aspects, first of all we do not compute all maximal cliques. We aim immediately towards high quality candidates rather than trying to recover them in a subsequent merging phase. Moreover, using an all-maximal-cliques approach may lead to a combinatorial time explosion in large PPIN, which we avoid.

1.6 CFinder

CFinder ([9]) is a fast program that implements the CPM (Clique Percolation Method) for graph clustering. The algorithm first finds all k-cliques in the input network, then associates two k-cliques if they share a common $(k-1)$-clique. The cluster produced by CFinder are the equivalence classes of this association relation. Note that the produced distinct clusters may share nodes, however the intersection of two clusters may contain $(k-2)$-cliques but never a $(k-1)$-clique. CFinder was one of the first clustering algorithms that could control the overlapping structure of the family of clusters it produces. The parameter k is critical and for biological applications a value in the range $k = 4, 5, 6$ is suggested.

1.7 RNSC

Restricted Neighborhood Search Clustering Algorithm (RNSC) ([10]) is a method for partitioning the nodes of a network into clusters that is loosely based on the tabu search meta-heuristic. In particular the meta-heuristic aims at optimizing a biologically inspired cost function, over the space of all possible partitions, starting from an initial random partition. For efficiency reasons the algorithm uses initially a quick and rough cost evaluation procedure, and in the finishing stages, a slower, more accurate cost evaluation procedure. Following the tabu-search paradigm, RNSC uses several strategies to escape local (non-optimal) minima of the objective cost function.

1.8 ProRank+

Prorank/Prorank+ ([11, 12]) use several edge pruning and filtering steps before applying a ranking step inspired by the well known Page-Rank algorithm in order to highlight candidate protein seeds. Afterwards, a complex is identified starting from a seed by using a spoke model. Finally highly overlapping complexes are removed in alternated duplicate-detection and cohesiveness-based-merge phases.

1.9 SPICI

SPICi ([13]) uses a greedy approach in which nodes of the network are ranked by their weighted degree, and are considered in that order as candidate cluster

seeds. A cluster is expanded starting from a seed by adding adjacent nodes that increase its weighted density. All elementary operations are supported by efficient data structures.

1.10 ClusterONE

ClusterONE ([14]) is a greedy method similar to SPICi but with significant differences. One difference is that in the cluster construction phase the algorithm uses both expansion and contraction operations. The computed clusters optimize a relative measure of cohesiveness that is essentially a ratio of the internal total weight of a cluster over the total weight of the edges that connect it to the complement graph. In a post-processing phase, highly overlapping clusters are merged.

2 Algorithms used for comparisons: experimental settings

We run our experiments on an Intel core i7 processor (4 cores) at 2.6GHz, with 16Gb RAM memory. The Operating System is Mac OS X 10.8.5. The HD holds 1Tb (with 128SSD). The software for *Core&Peel* has been developed in Python 2.7. without any optimization to exploit the multi-core features of the processor.

2.1 MCL

The MCL C code was downloaded from:
`http://micans.org/mcl/src/mcl-12-068.tar.gz.`

The MCL algorithm has one main parameter that is the *inflation* whose value affects the cluster granularity. Smaller values will tend to result in a very coarse clustering while larger values tend to result in a fine-grained clustering. We have tested the inflation parameter in the range from 1.5 to 4.0 in steps of 0.1 (26 steps).

2.2 MCL-CAw

The MCL-CAw code was downloaded from
`https://sites.google.com/site/mclcaw/.`
MCL-CAw takes as input a weighted PPIN, and a MCL output file. We use the 26 MCL files generated in the MCL runs with different values of the inflation parameter for each PPIN, and we modified the input PPI network files by adding a weight (set to one) to each protein interactions. MCL-CAw has also two parameters α and β, which we set to the default values $\alpha = 1.0$ and $\beta = 0.40$ (strongly recommended in the original paper).

The Biogrid PPINs proved too large and we got an execution error (segmentation-fault) when feeding them to MCL-CAw. The code performed well in Biogrid homo sapiens PPIN after removing the UBC protein. Segmentation-faults were also obtained on some runs (but not all) for String PPIN. The performance statistics were compiled for the completed runs.

2.3 COACH

The COACH windows executable was downloaded from
`http://www1.i2r.a-star.edu.sg/~xlli/coach.zip.`
We run COACH with the default parameters. We do not list results for the String-hs PPIN since COACH did not terminate on this data set after 18h computation.

2.4 MCODE

MCODE was re-implemented in Python 2.7 by us because the standard Cytoscape MCODE Plugin could not be applied to large graphs. We validated our implementation on small examples and obtained the same result as the original code. MCODE has several parameters for whose meaning we refer to the Cytoscape MCODE Plugin online documentation.

We set the *haircut* and the fluff parameter flags to on/off, the *Node Density Cutoff* to values in $(0.8, 0.6, 0.5, 0.4, 0.2)$, *Node Score Cutoff* to values in $(0.1, 0.2, 0.3, 0.4, 0.5)$, and *maxdepth* to values in $(1000, 10000)$. The *K-Core* parameter is set to the default value 2. A total of 72 different configurations were tested for each dataset.

2.5 CMC

The CMC windows executable code was downloaded from:

`http://www.comp.nus.edu.sg/~wongls`
`/projects/complexprediction/CMC-26may09/.`

CMC expects as input a PPI file with weighted interactions. We used the program provided within the downloaded CMC code to weight the PPI interactions according to three different weighting methods: CD-Distance, Adjusted CD-distance, and FS-Weight. We also tested the case of all weights set to 1.0.

The *minimum cluster size* parameter set to values 3, 4, 6 and 8, the *overlap-threshold 0.5*, and *merge-threshold* to 0.25 (default parameters). Thus we tested a total of 16 configurations on each PPIN.

2.6 CFinder

We downloaded the CPM implementation in C++ code from the the SNAP Software Package:
`http://snap.stanford.edu/snap/description.html`
The main control parameter is the value k of the basic cliques produced. We run experiments with $k = \{3, 4, 5, 6, 7, 8\}$.

2.7 RNSC

The RNSC C code was downloaded from:

`www.cs.utoronto.ca/~juris/data/rnsc/`

Additional details on the usage of the software are in:

`http://www.cs.utoronto.ca/~juris/data/`
`rnsc/rnsc_techreport.ps`

The algorithm has several parameters (seven) that mainly control the tabu search part of the algorithm. A study in [2] performed extensive testing on RNSC and propose two settings, one optimizing accuracy and one optimizing separation. We used these two settings, and the default one.

Data set	min size	min density	filter type	max sep.
DIP-y	4	0.7	0	0.9
BioGrid-y	7	1.0	1	0.8
String-y	6	0.9	1	0.9
BioGrid-hs	5	1.0	0	0.8
BioGrid-hs-UBC	5	1.0	2	0.9
String-hs	5	1.0	0	0.8

Table 1 Parameters for which the best F1-measure of Core&Peel is attained.

2.8 ProRank+

The ProRank+ Python code was downloaded from:
`http://faculty.uaeu.ac.ae/nzaki/Research.htm`

The procedure takes in input the PPIN file, and has no other parameters.

2.9 SPICi

The SPICi C code was downloaded from:
`http://compbio.cs.princeton.edu/spici/`

SPICi was run with the *min-size parameter* $s = \{3, 4, 5, 6, 8\}$ and the density parameters $c = \{1.0, 0.8, 0.5\}$ for String and Biogrid data, and $c = \{0.8, 0.7, 0.6, 0.5\}$ for DIP data. The *min support* parameters is set to the default 0.5. The *file type parameter* m is set to 2, which is optimized for large sparse graphs.

2.10 ClusterONE

The ClusterONE java executable code was downloaded from:
`http://www.paccanarolab.org/cluster-one/`

it was run with the min-size parameter $s = \{3, 4, 5, 6, 8\}$, and the density parameters $c = \{1.0, 0.9, 0.8\}$ for String and Biogrid data. For DIP data, which is sparser, we used $c = \{0.8, 0.7, 0.6, 0.5\}$.

3 Parameters settings for Core & Peel

Core&Peel has 4 main parameters , the min size, the min density, the filter type and the max Jaccard separation. Table 1 gives the parameter configurations for which *Core&Peel* attains the best results of the F1 measure. The best f-measure is attained with a target size close to the mean size of the known complexes (about 6), and a value of the density parameter c close to the actual prevalent density of the sought complexes in the given graph. This indicates that, in an operational situations, by measuring samples of known complexes in the network, we have some guidelines for choosing the parameters likely to attain the best performance for *Core&Peel*. These guidelines are also in line with the expected influence of each parameter on the output quality.

4 Handling of small complexes in comparative evaluation of different methods

Many of the tools used in this evaluation (e.g. SPICi, ClusterOne, Core&Peel, CMC) require the user to give explicitly a minimum size to the cardinality of the clusters

returned as output. Cfinder does not explicitly set a min-size parameter, but the value of the minimum clique k used to define the clusters it can produce constitutes a lower bound also to the cluster size. Other methods (e.g. MCL, MCL-caw, COACH, MCODE, RNSC, ProRank+) do not require an explicit minimum size value to be set. This variety of approaches may derive from several considerations. Methods of the first type often rely on density-based definitions that become vacuous for the cases of size n=1 (a single vertex of the graph), or n=2 (a single edge of the graph). Moreover there may be an assumption that the method is more reliable to detect larger complexes, rather than smaller ones. Also another reasonable assumption is that the user may be interested in using such prediction method just for relatively large objects, since for smaller ones, alternative approaches could be used. Naturally for the second group of methods these considerations may or may not hold. However, since both the golden standard data sets CYC2008 and CORUM contain a large fraction of small complexes, proper handling of them becomes critical for a fair evaluation. The guiding principle of our evaluation is that no method should be penalized for making legitimate assumptions, nor it should be penalized for performing well even when these assumptions are not fulfilled.

Thus we proceed as follows. Each Golden Standard set G is stratified by considering sets G_i of its elements of size $\geq i$, for $i = 1, 2, 3$. Each Predicted set P is also stratified by considering sets P_j of its elements of size $\geq j$, for $j = 1, 2, 3$. For each performance function F we define $F(G, P)$ to be the maximum of $F(G_i, P_j)$ for all choices of $i = 1, 2, 3$ and $j = 1, 2, 3$ (9 cases). Note that we do evaluate also pair of stratified sets made with different min size parameters.

5 Weighted vs. unweighted PPI Networks

In the supplementary materials of [14][Figure 1] four PC prediction algorithms are compared when executed on four weighted networks and on the same four networks with the weights removed (i.e all weights set to 1). In these experiments it is shown that the use of weights helps, however the improvement is not dramatic and it can be quantified as a relative improvement of about (10%) of the aggregated score used in [14].

The edge weights in Weighted PPI Networks can have different sources and meanings. At the simplest level the weight of a PPI derived from a single high throughput experiment can reflect some physical measurement associated to the specific technology employed. At a higher level, such a weight may be a measure of confidence on the existence of a PPI that derives from merging many different elementary sources of evidence, like in [15] and in [16]. Principled merging of PPI weights coming from different, heterogeneous experiments and data sources is still quite an open research problem. When large networks are built as an ensemble of smaller networks, often weighting information is not carried over to the larger networks. Therefore, even

if methods for weighted networks are more general, methods handling unweighted networks are also useful in the context of large networks analysis.

6 Distributions of features of the test data

6.1 Figure synopsys

In this section we report the distributions of basic features for our test graphs, and for the known protein complexes embedded in these graphs. We report the distributions of node degrees (Figures 1, 9, 17, 25, 33), node core numbers (Figures 2, 10, 18, 26, 34), and node clustering coefficients (Figures 3, 11, 19, 27, 35).

For the known protein complexes considered as subgraphs of the test graphs we report the distribution of complex sizes (Figures 4, 12, 20, 28, 36), complex average degrees (Figures 5, 13, 21, 29, 37), and complex densities (Figures 6, 14, 22, 30, 38). Moreover, we give scatter-ball plots of the joint distribution of core number and degree of the nodes (Figures 7, 15, 23, 31, 39), and of the joint distribution of core number and clustering coefficient of the nodes (Figures 8, 16, 24, 32, 40).

For the biogrid homo sapiens dataset without the UBC protein the distributions are qualitatively unchanged, except for the removal of an isolated point of maximum degree (degree about 10,000 as see in figure 25), the new maximum degree node in the residual network has degree about 2000; moreover about 1400 nodes become isolated nodes (data not shown).

6.2 Discussion of figures

The node degree distributions in all the five graphs have qualitatively a power-law shape, as it is typical for many biological networks of this kind [17] (Figures 1, 9, 17, 25, 33).

The scatter plots of the core number vs node degree show a dual regime of correlation for dip (Fig.7), biogrid-yeast (Fig. 15), string-yeast (Fig. 23), biogrid-hs (Fig. 31), and string-hs (Fig. 39), with a slowly increasing part of the plot where the core number increases with the degree, and a saturation point where the same maximal core number corresponds to a wide range of node degrees. For string-hs (Fig. 39) there is also an isolated component in the plot of higher degree and higher core number.

The same type of plot where we consider the correlation of core number and clustering coefficient (Fig.8, 16, 24, 32, 40) shows a more varied range of behaviors. For dip data (Fig.8) and biogrid-yeast data (Fig. 16) the core number is saturated even at a low core coefficient value about 0.6 (resp. 0.4). While for string-yeast, biogrid-hs and string-hs data there is a spread of core number values for a fixed correlation coefficient value (about 0.8 and 1.0). This shows that the use of core numbers indeed uncovers and discriminates hidden structures that are not evident when measured via the clustering coefficients

The size and average degree distributions of the known complexes (Fig. 4, 5, 12, 13, 20, 21, 28, 29, 36, 37) have all a power-law shape (just somewhat more irregular for the smaller dip graph). The complex density distributions (Fig. 6, 14, 22, 30, 38) highlight the trend towards density saturation for yeast graphs as they become larger. For human data (Fig 30, 38) two quite similar distributions are obtained with the two dense graphs (biogrid and string).

Overall, these plots highlight both similarities and differences among the data set used. As a sanity check these tests do not reveal any pathological or unexpected behaviour for the class of large PPI networks, thus we consider them rather typical of networks one may want to consider in practice.

6.3 DIP-yeast graph

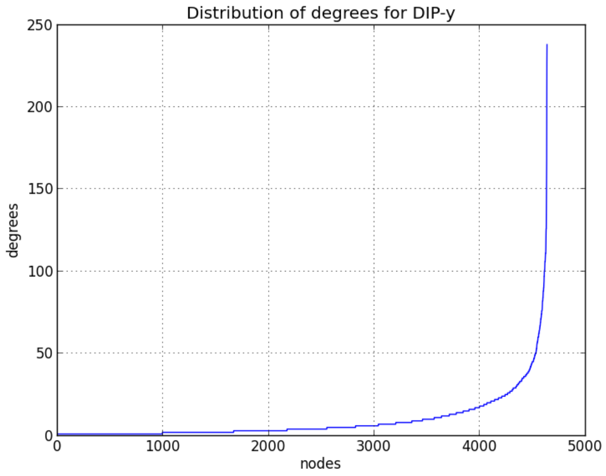

Figure 1 Degree distribution for nodes in the DIP-yeast graph.

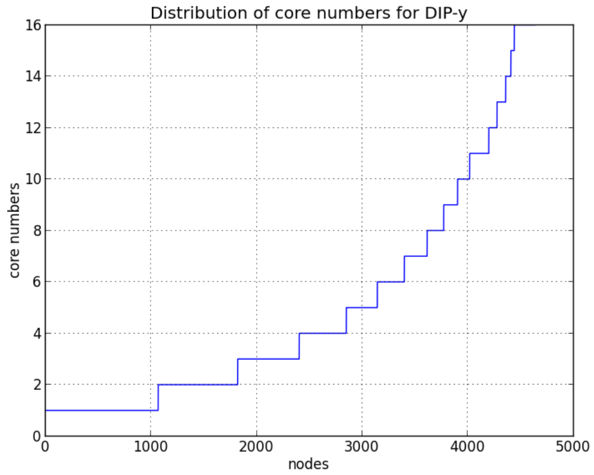

Figure 2 Core number distribution for nodes in the DIP-yeast graph.

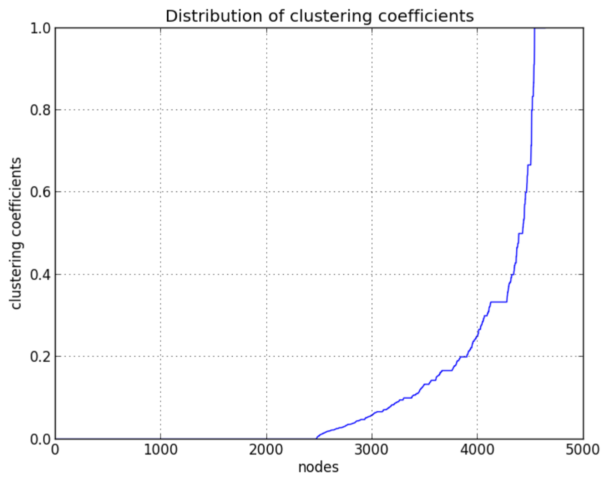

Figure 3 Clustering Coefficient distribution for nodes in the DIP-yeast graph.

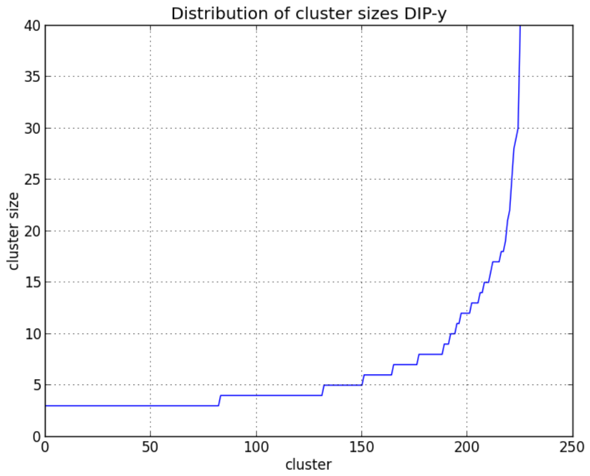

Figure 4 Size distribution for protein complexes (≥ 3) in the DIP-yeast graph.

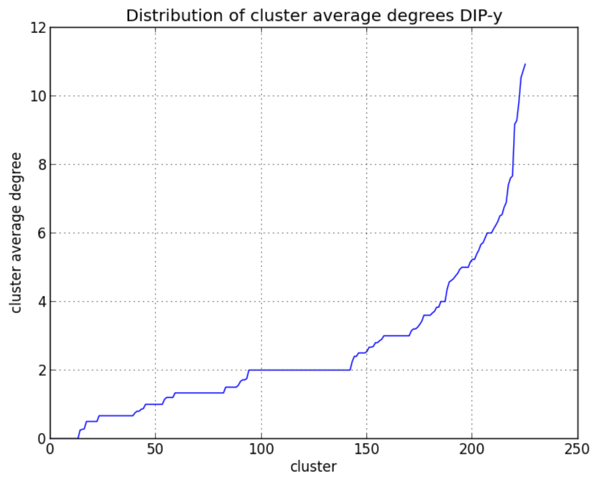

Figure 5 Average degree distribution for protein complexes (≥ 3) in the DIP-yeast graph.

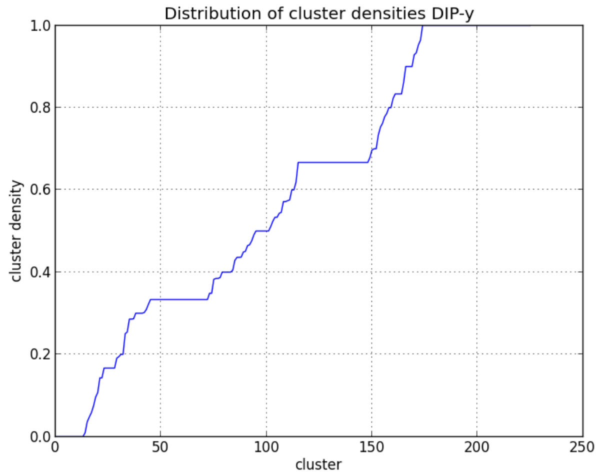

Figure 6 Density distribution for protein complexes (≥ 3) in the DIP-yeast graph.

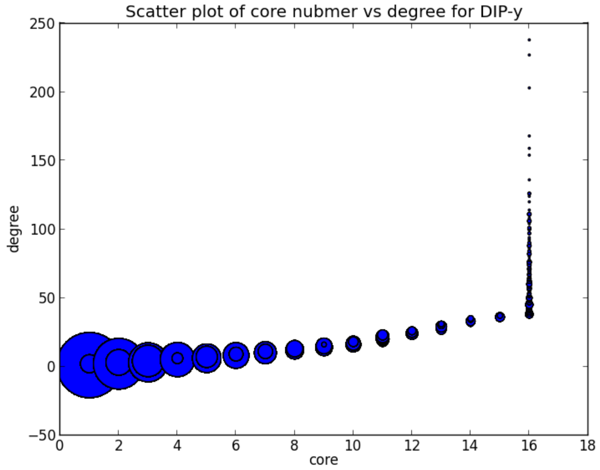

Figure 7 Scatter-ball plot of core number vs degree of nodes in the DIP-yeast graph. Radius of balls is proportional to the number of nodes having a given pair of values (coordiantes of the center of a ball).

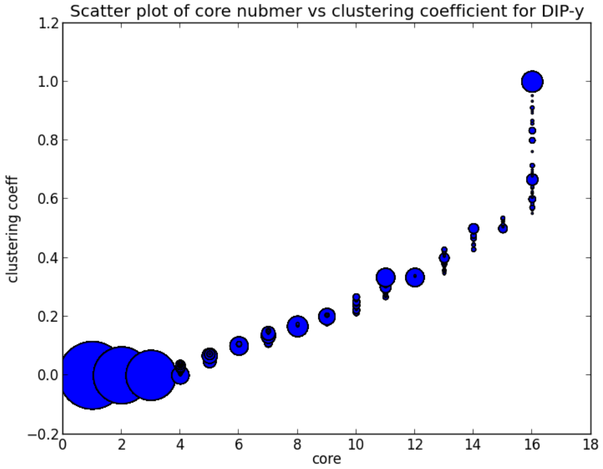

Figure 8 Scatter-ball plot of core number vs clustering coefficient of nodes in the DIP-yeast graph. The radius of balls is proportional to the number of nodes having a given pair of values (coordinates of the center of a ball).

6.4 Biogrid-yeast graph

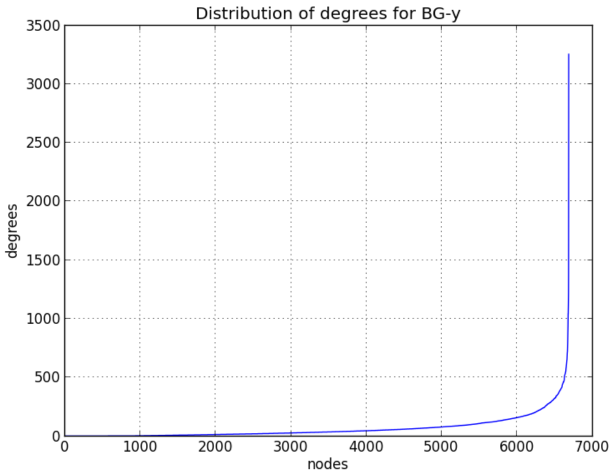

Figure 9 Degree distribution for nodes in the Biogrid-yeast graph.

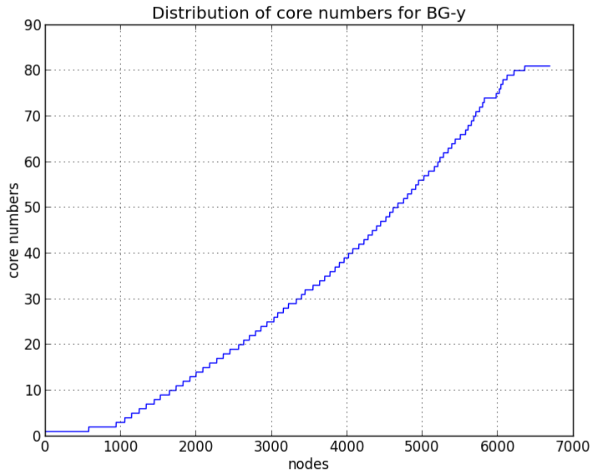

Figure 10 Core number distribution for nodes in the Biogrid-yeast graph.

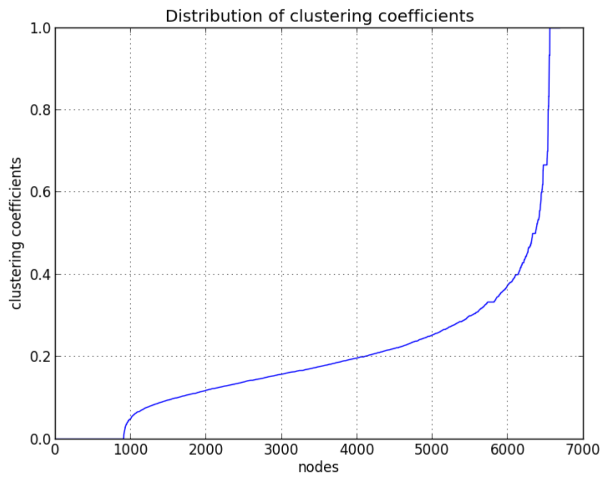

Figure 11 Clustering Coefficient distribution for nodes in the Biogrid-yeast graph.

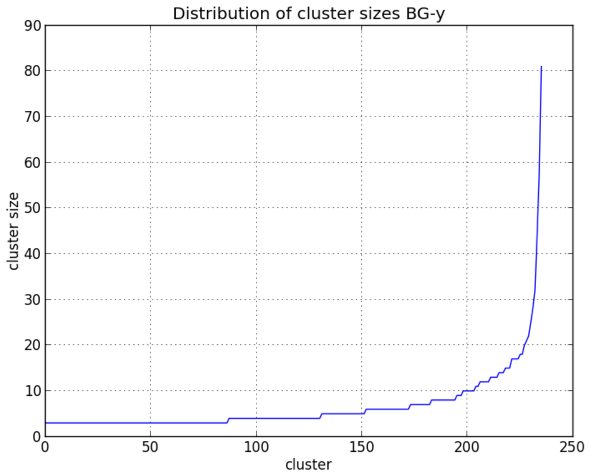

Figure 12 Size distribution for protein complexes (≥ 3) in the Biogrid-yeast graph.

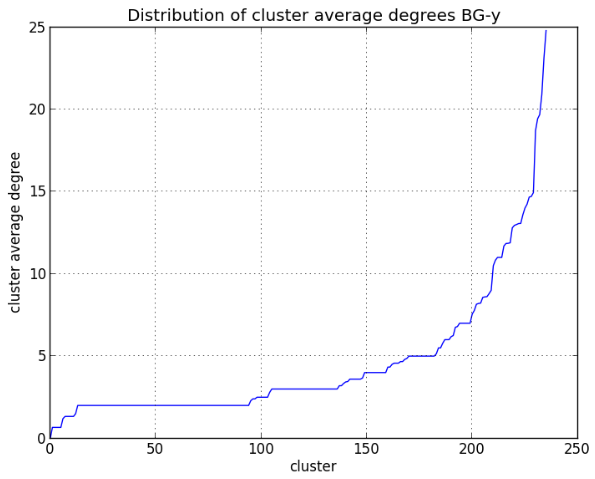

Figure 13 Average degree distribution for protein complexes (≥ 3) in the Biogrid-yeast graph.

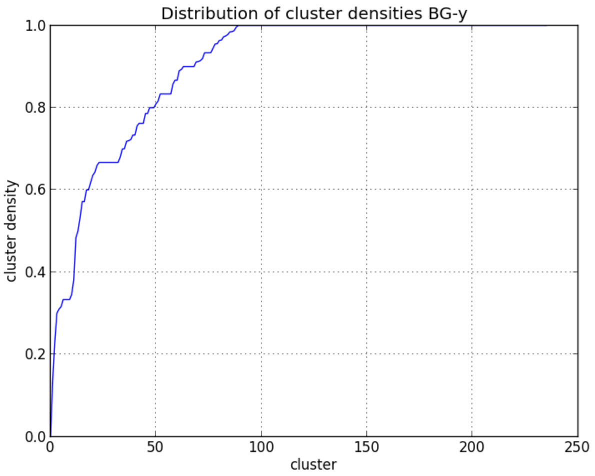

Figure 14 Density distribution for protein complexes (≥ 3) in the Biogrid-yeast graph.

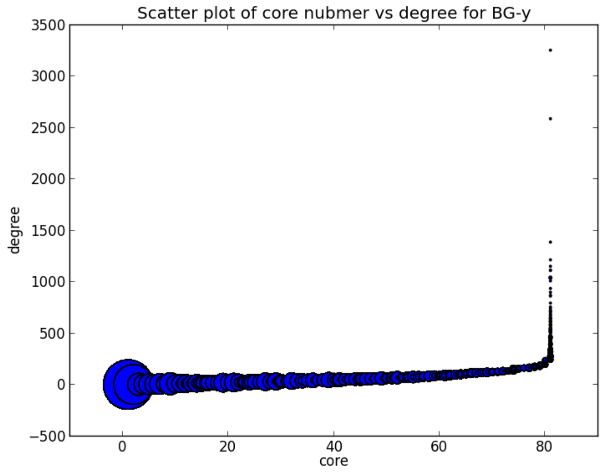

Figure 15 Scatter-ball plot of core number vs degree of nodes in the Biogrid-yeast graph. Radius of balls is proportional to the number of nodes having a given pair of values (coordiantes of the center of a ball).

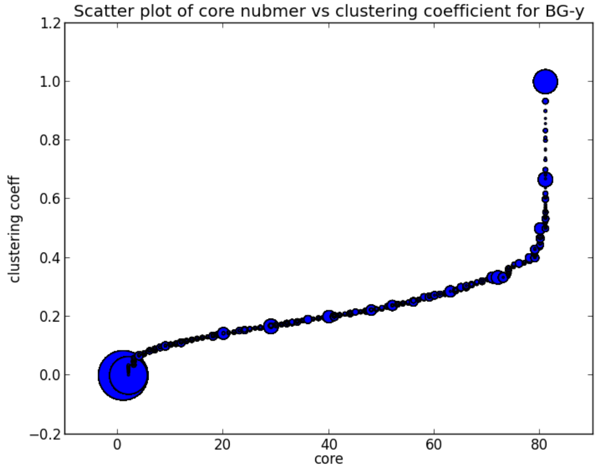

Figure 16 Scatter-ball plot of core number vs clustering coefficient of nodes in the Biogrid-yeast graph. The radius of balls is proportional to the number of nodes having a given pair of values (coordinates of the center of a ball).

6.5 String-yeast graph

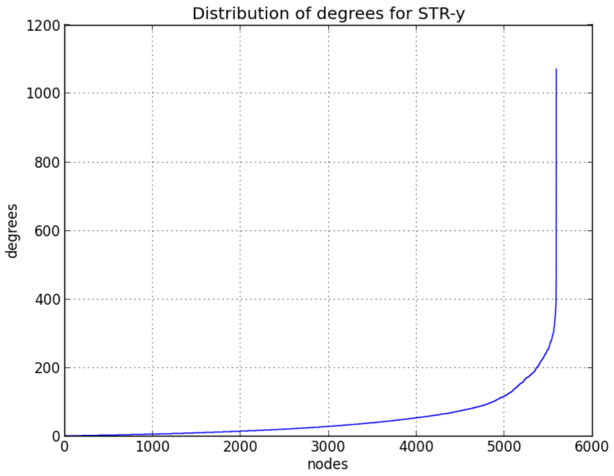

Figure 17 Degree distribution for nodes in the String-yeast graph.

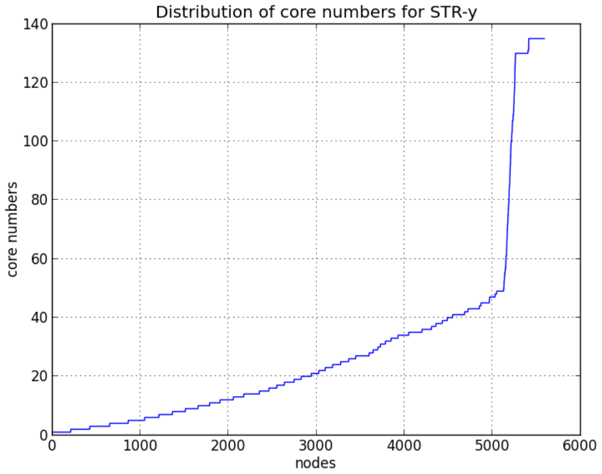

Figure 18 Core number distribution for nodes in the String-yeast graph.

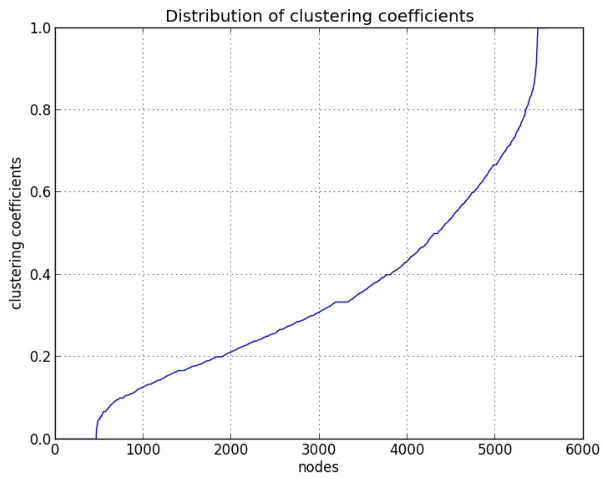

Figure 19 Clustering Coefficient distribution for nodes in the String-yeast graph.

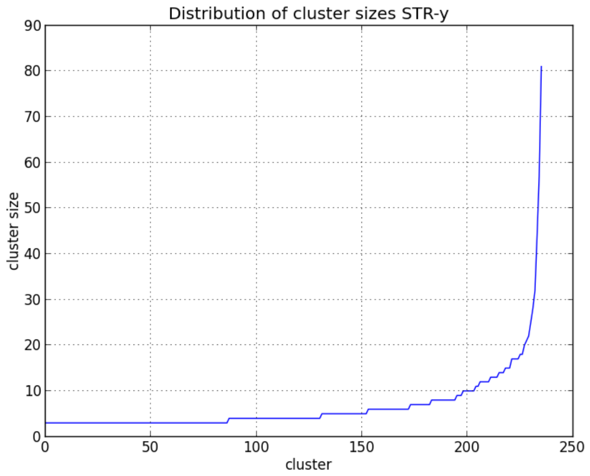

Figure 20 Size distribution for protein complexes (≥ 3) in the String-yeast graph.

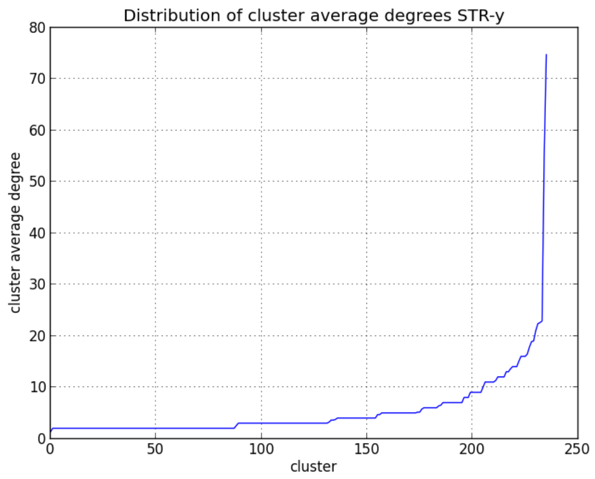

Figure 21 Average degree distribution for protein complexes (≥ 3) in the String-yeast graph.

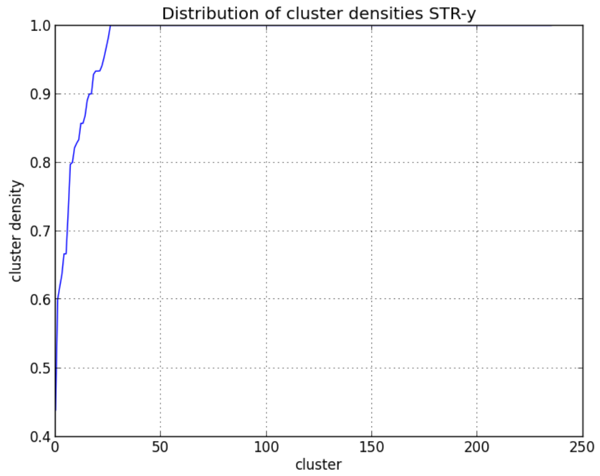

Figure 22 Density distribution for protein complexes (≥ 3) in the String-yeast graph.

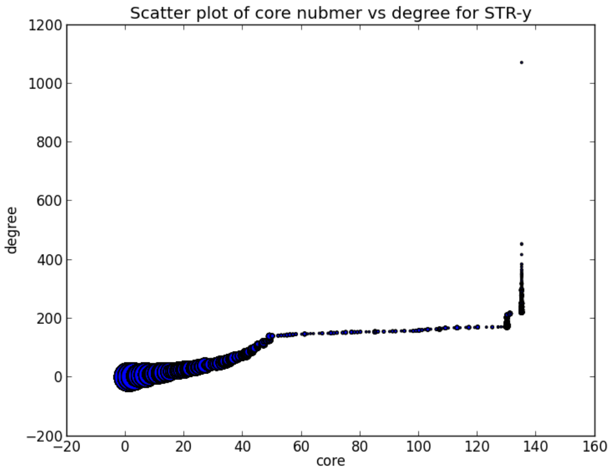

Figure 23 Scatter-ball plot of core number vs degree of nodes in the String-yeast graph. Radius of balls is proportional to the number of nodes having a given pair of values (coordiantes of the center of a ball).

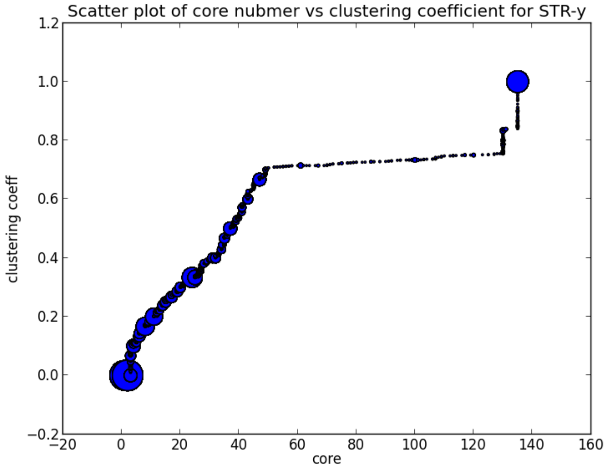

Figure 24 Scatter-ball plot of core number vs clustering coefficient of nodes in the String-yeast graph. The radius of balls is proportional to the number of nodes having a given pair of values (coordinates of the center of a ball).

6.6 Biogrid-homo sapiens graph

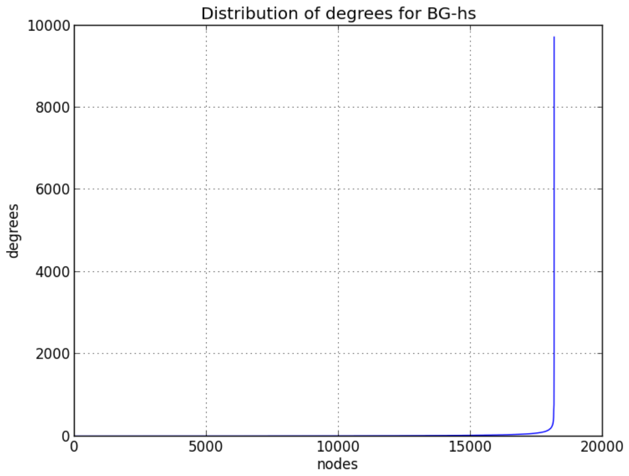

Figure 25 Degree distribution for nodes in the Biogrid-homo sapiens graph.

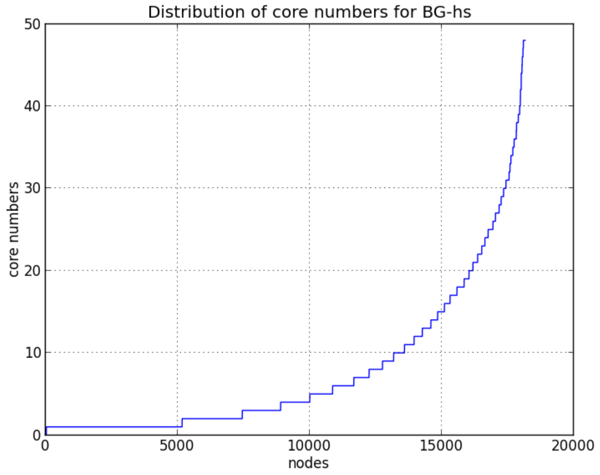

Figure 26 Core number distribution for nodes in the Biogrid-homo sapiens graph.

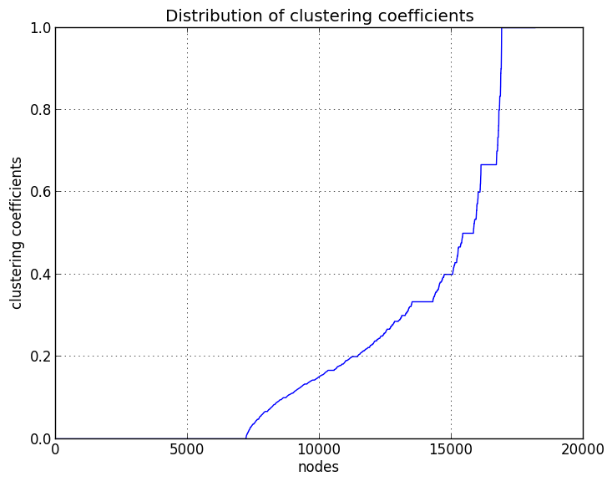

Figure 27 Clustering Coefficient distribution for nodes in the Biogrid-homo sapiens graph.

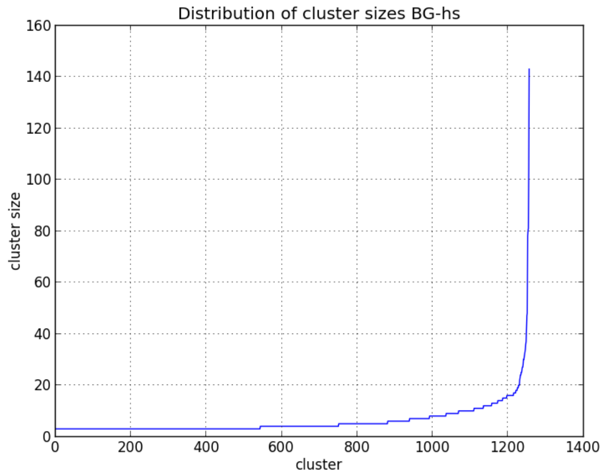

Figure 28 Size distribution for protein complexes (≥ 3) in the Biogrid-homo sapiens graph.

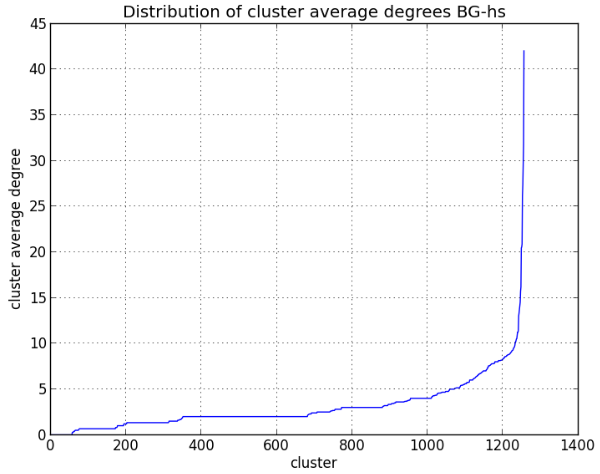

Figure 29 Average degree distribution for protein complexes (≥ 3) in the Biogrid-homo sapiens graph.

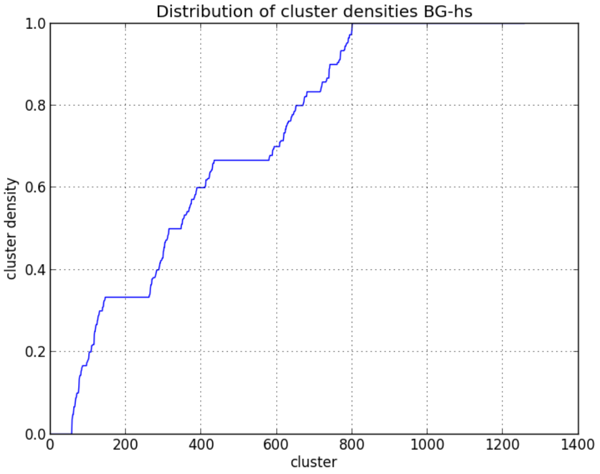

Figure 30 Density distribution for protein complexes (≥ 3) in the Biogrid-homo sapiens graph.

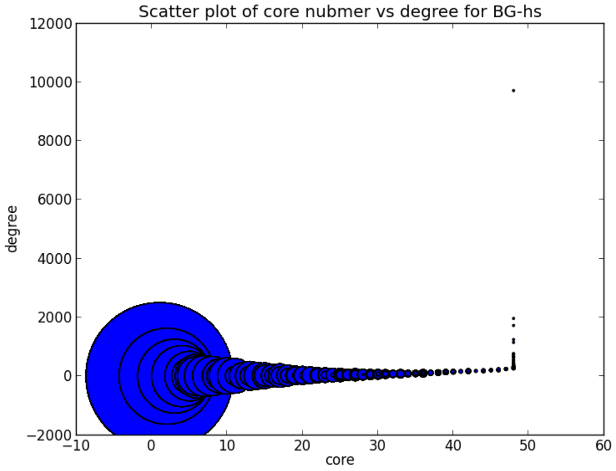

Figure 31 Scatter-ball plot of core number vs degree of nodes in the Biogrid-homo sapiens graph. Radius of balls is proportional to the number of nodes having a given pair of values (coordiantes of the center of a ball).

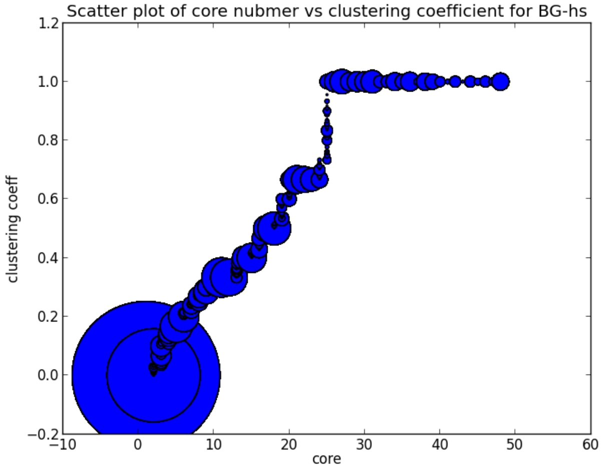

Figure 32 Scatter-ball plot of core number vs clustering coefficient of nodes in the Biogrid-homo sapiens graph. The radius of balls is proportional to the number of nodes having a given pair of values (coordinates of the center of a ball).

6.7 String-homo sapiens graph

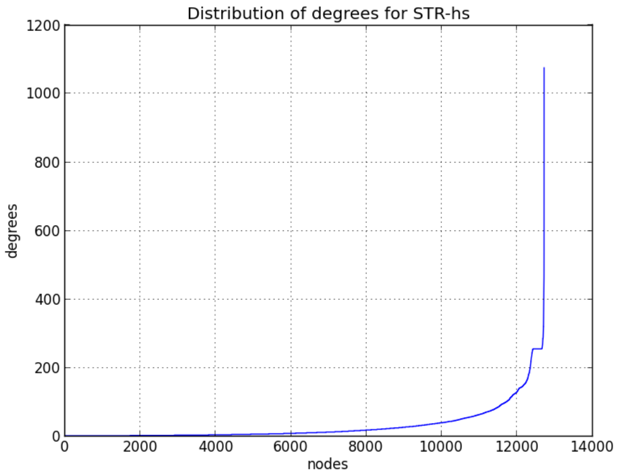

Figure 33 Degree distribution for nodes in the String-homo sapiens graph.

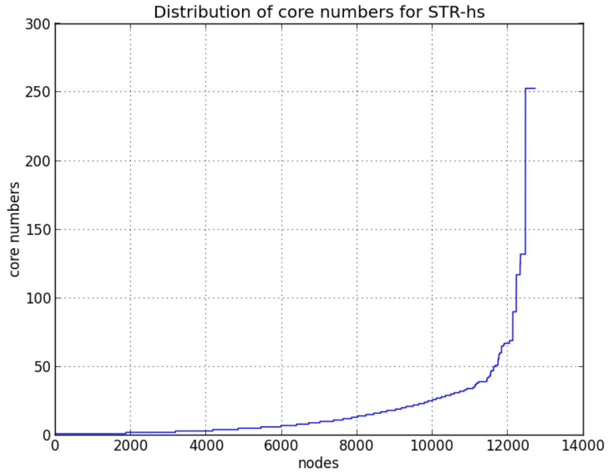

Figure 34 Core number distribution for nodes in the String-homo sapiens graph.

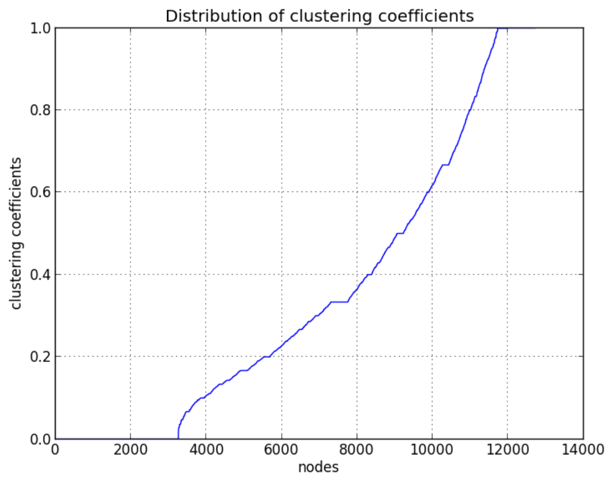

Figure 35 Clustering Coefficient distribution for nodes in the String-homo sapiens graph.

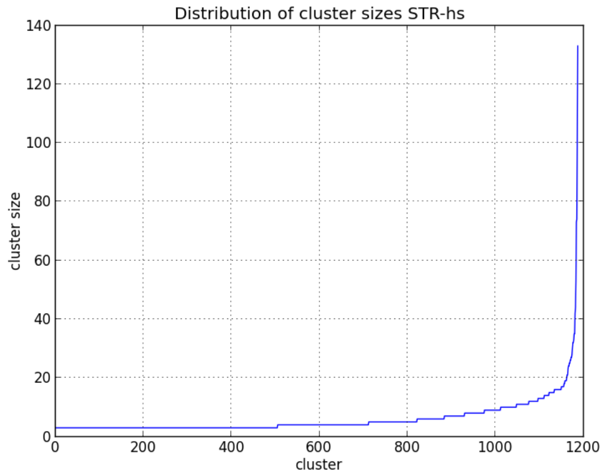

Figure 36 Size distribution for protein complexes (≥ 3) in the String-homo sapiens graph.

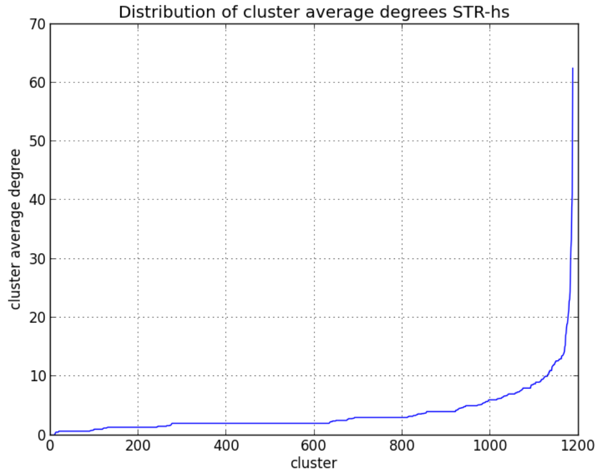

Figure 37 Average degree distribution for protein complexes (≥ 3) in the String-homo sapiens graph.

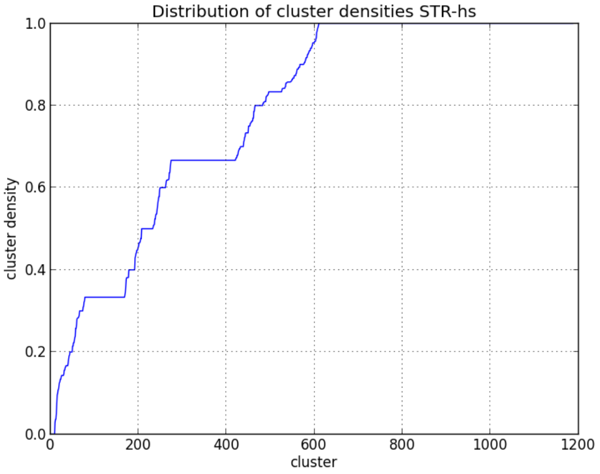

Figure 38 Density distribution for protein complexes (≥ 3) in the String-homo sapiens graph.

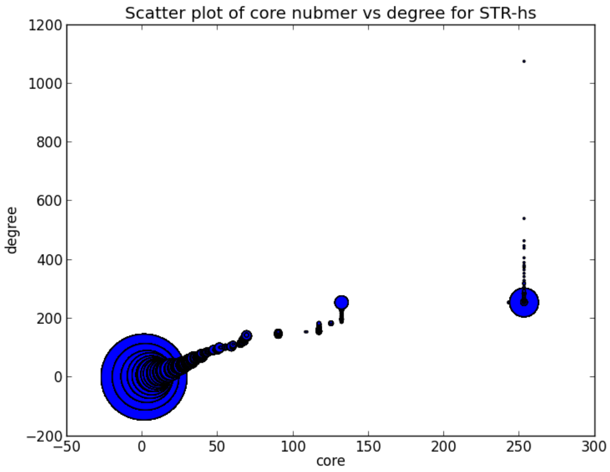

Figure 39 Scatter-ball plot of core number vs degree of nodes in the String-homo sapiens graph. Radius of balls is proportional to the number of nodes having a given pair of values (coordiantes of the center of a ball).

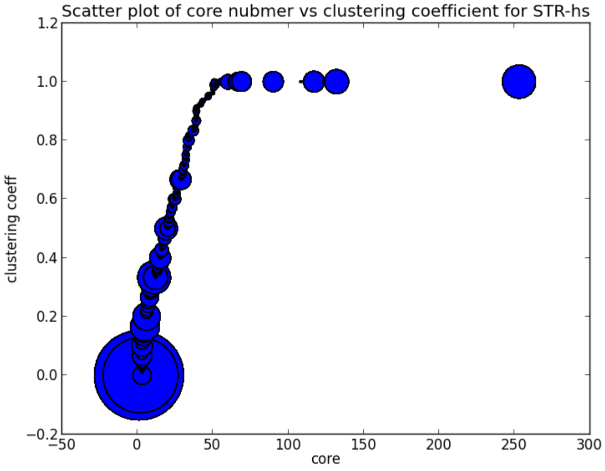

Figure 40 Scatter-ball plot of core number vs clustering coefficient of nodes in the String-homo sapiens graph. The radius of balls is proportional to the number of nodes having a given pair of values (coordinates of the center of a ball).

Data set	min size	exp. #	stnd dev #	observed #	p-value	FDR
ER-DIP-y	4	3.3	1.83	1360	0.0	0.0026
ER-BioGrid-y	4	77.0	8.74	4902	0.0	0.015
ER-String-y	4	14.6	2.52	3241	0.0	0.004
ER-BioGrid-hs	3	579.4	25.2	10276	0.0	0.056
ER-Biogrid-UBC-hs	3	463.9	22.21	8241	0.0	0.056
ER-String-hs	4	0.3	0.57	3851	0.0	0.00007

Table 2 ER. Statistics of the found clusters in a PPIN versus the distribution of clusters found in random ER graphs of same size, using the parameters optimizing the f-measure. P-values are estimated via conversion form the z-scores of 20 independent samples.

7 Random graphs

One may be interested in the fraction of the predicted clusters we find in our test graphs that can be attributed to the background clustering properties of a random graph with the same number of nodes and edges. In order to explore this issue we used the python package *NetworkX* (https://networkx.github.io/) that generates several classes of random graphs. In [18] several random graph models are evaluated for their ability to replicate known features of real PPI graphs. In that study the Erdős–Rényi (ER) model is shown to be the random graph model less able to mimic PPI graphs, therefore we adopt it as our baseline model. We adopt the two stage multiple hypothesis test proposed in [19, 20] to bound the false discovery rate (FDR) associated with the identified complexes.

The first test is as follows. We evaluate the p-value associated with the rejection of the null hypothesis that the observed number of complexes of size greater or equal to a threshold value s falls within the size distribution generated by the algorithm applied to a random ER graph with the same number of nodes and arcs. The second test estimates the False Discovery Rate we have when we accept all computed clusters of size greater or equal to s as significant (i.e. the expected number of null hypothesis we wrongly reject).

We run *Core&Peel* with the parameter configuration giving the maximal value of the F-measure (see Table 1), but with minimum size set to 3, and we report the size class with the associated FDR that is closest to the reference value 0.05. The values of Table 1 are generated with 20 random graph samples.

Comparing the threshold sizes values in Table 2 with the min size parameters of Table 1 we notice that the min size parameter always falls within the region of reasonable FDR values (about or below 0.05). The procedure outlined in this section may give a guidance in setting a proper min size parameter value in situations when no other hint is available.

8 Asymptotic complexity of Core & Peel

Here we give a proof sketch of the asymptotic time complexity bound of each phase in terms of the number of vertices n, and the number of edges m of the input graph G, as well as of other measures on the graph G. We neglect polylogarithmic terms such as those one could have from sorting, or using dictionary data structures.

Phase I. The computation of the core decomposition using the algorithm of [21] is extremely fast, taking linear time: $O(n+m)$, and uses very simple data structures. The calculation of $CC(v)$ for each node v also takes linear time $O(n+m)$. The

lexicographic sorting can be done in $O(n+m)$ using bucket-sorting as basic sorting algorithm.

Phase II. This phase is dominated by the time needed to compute for each vertex v its relevant set of neighbors at distance 1, $N_{C(v)}(v)$, and the induced graph $G[N_{C(v)}(v)]$. Each pair of vertices joined by an edge in $G[N_{C(v)}(v)]$ form by construction a triangle with v in G. Therefore the problem is easily solved when we are able to find and list explicitly all triangles in a graph. The algorithm of [22] reports all triangles in an undirected graph in time $O(a(G)m)$, where the measure $a(G)$ is the so called *arboricity* of the graph G. This is the minimum number of edge-disjoint spanning forests into which G can be decomposed. This number can also be well approximated with the maximum of the average degree of any subgraph in G. Interestingly in PPIN, which are rather sparse graphs, this number is quite small. The arboricity of a graph is bounded from above by the smallest number between $\sqrt{2m+n}/2$ and the maximum degree of the graph [22].

Phase III. Having a suitable representation of $G[N_{C(v)}(v)]$ for each v, the peeling operations take time $O(1)$ for each removed edge incident to a node of minimum degree. The nodes of minimum degree under dynamic updates of the graph can be found in time $O(1)$ using the data structures of [21]. The update time is proportional to the number of deleted edges in each iteration. Therefore the cost of the peeling phase is at most the size of the input sub-graph (for which the size bound described above holds). More care has to be used in evaluating and updating the vertex measure $S(v)$. However we notice that $S(v)$ is used only to break ties during the peeling procedures, and in practice it is invoked only on a small fraction of the peeling iterations. In this setting a direct calculation of $S(v)$, in time proportional to the current degree of v, is done only when it is needed to solve ties.

Phase IV. The total size of all the dense subsets found is smaller than the size of the neighbor graphs generated in Phase II, for which we have an upper bound $O(a(G)m)$. The lexicographic sorting can be done in (almost) linear time in the size of the objects to be sorted (up to small polylogarithmic terms). Therefore eliminating duplicates has a time complexity comparable with that of the previous phases.

For the removal of included sets we denote with $\{S_i\}$ the family of sets of nodes generated by phase III, after the removal of duplicates, and with $\{s_i\}$ the center node corresponding to each set. The time complexity is dominated by the total time to perform the BFS, which is proportional to $\sum_{i \neq j} |S_i \cap S_j|$. Next, we will use properties of the graph G and some mild hypothesis on the family of sets produced by phase III in order to upper bound the time complexity of this phase. We assume that for any vertex, the number of sets S_i that include that vertex is at most a constant number c.

1) Consider the contributions of all pairs i, j for which $|S_i \cap S_j| = 1$, and neither center belong to the intersection. In this case, this class of pairs is partitioned by the associated single node in the intersection. On the other hand since each node can be associated to at most c such sets, therefore it is associated to at most c^2 such pairs. The total contribution of these pairs is thus $O(c^2 n) = O(n)$.

2) Consider the contributions of all pairs i, j for which $|S_i \cap S_j| \geq 2$, and neither center belong to the intersection. In this case the centers s_i and s_j and two distinct

elements of the $S_i \cap S_j$ form a quadrilateral in G. Next, we will use the compact data structure in [22] which represents uniquely all quadrilaterals in G in a data structure of size $O(a(G)m)$. This data structure is a list of triplets of the form (s_i, s_j, L_{ij}) where $L_{ij} \subseteq S_i \cap S_j$, or of the form (v_x, v_y, S_{xy}), where S_{xy} is a set of centers of sets that have both v_x and v_y in all the their pairwise mutual intersection of the associated sets. For the first type of triplets, we notice that the sum of the sizes of all lists L_{ij} is bounded by $O(a(G)m)$. For the second type of triplet, each list S_{xy} contributes at most $|S_{xy}|^2$ units to the final count, but $|S_{xy}| < c$ by hypothesis, therefore, also this second type of triplet encodes at most $O(c^2 a(G)m) = O(a(G)m)$ contributions.

3) If $|S_i \cap S_j| >= 2$ and either s_i or s_j or both belong to $S_i \cap S_j$, then s_i, s_j and any distinct node in $S_i \cap S_j$ form a triangle. This case also is bounded by $O(a(G)m)$ by an argument in [22].

References

1. Van Dongen, S.: Graph clustering via a discrete uncoupling process. SIAM J. Matrix Anal. Appl. **30**(1), 121–141 (2008). doi:10.1137/040608635
2. Brohee, S., van Helden, J.: Evaluation of clustering algorithms for protein protein interaction networks. BMC Bioinformatics **7**(1), 488 (2006). doi:10.1186/1471-2105-7-488
3. Srihari, S., Ning, K., Leong, H.: Mcl-caw: a refinement of mcl for detecting yeast complexes from weighted ppi networks by incorporating core-attachment structure. BMC Bioinformatics **11**(1), 504 (2010). doi:10.1186/1471-2105-11-504
4. Wu, M., Li, X., Kwoh, C.K., Ng, S.-K.: A core-attachment based method to detect protein complexes in ppi networks. BMC Bioinformatics **10** (2009)
5. Seidman, S.B.: Network structure and minimum degree. Social Networks **5**(3), 269–287 (1983). doi:10.1016/0378-8733(83)90028-X
6. Bader, G., Hogue, C.: An automated method for finding molecular complexes in large protein interaction networks. BMC Bioinformatics **4**(1), 2 (2003). doi:10.1186/1471-2105-4-2
7. Liu, G., Wong, L., Chua, H.N.: Complex discovery from weighted ppi networks. Bioinformatics **25**(15), 1891–1897 (2009). doi:10.1093/bioinformatics/btp311. http://bioinformatics.oxfordjournals.org/content/25/15/1891.full.pdf+html
8. Tomita, E., Tanaka, A., Takahashi, H.: The worst-case time complexity for generating all maximal cliques and computational experiments. Theor. Comput. Sci. **363**(1), 28–42 (2006)
9. Adamcsek, B., Palla, G., Farkas, I.J., Derényi, I., Vicsek, T.: Cfinder: Locating cliques and overlapping modules in biological networks. Bioinformatics **22**(8), 1021–1023 (2006). doi:10.1093/bioinformatics/btl039
10. King, A.D., Pržulj, N., Jurisica, I.: Protein complex prediction via cost-based clustering. Bioinformatics **20**(17), 3013–3020 (2004). doi:10.1093/bioinformatics/bth351
11. Zaki, N., Berengueres, J., Efimov, D.: Detection of protein complexes using a protein ranking algorithm. Proteins: Structure, Function, and Bioinformatics **80**(10), 2459–2468 (2012). doi:10.1002/prot.24130
12. Hanna, E., Zaki, N.: Detecting protein complexes in protein interaction networks using a ranking algorithm with a refined merging procedure. BMC Bioinformatics **15**(1), 204 (2014). doi:10.1186/1471-2105-15-204
13. Jiang, P., Singh, M.: Spici: a fast clustering algorithm for large biological networks. Bioinformatics **26**(8), 1105–1111 (2010). doi:10.1093/bioinformatics/btq078. http://bioinformatics.oxfordjournals.org/content/26/8/1105.full.pdf+html
14. Nepusz, T., Yu, H., Paccanaro, A.: Detecting overlapping protein complexes in protein protein interaction networks. Nature Methods **9**, 471–472 (2012)
15. Jansen, R., Yu, H., Greenbaum, D., Kluger, Y., Krogan, N.J., Chung, S., Emili, A., Snyder, M., Greenblatt, J.F., Gerstein, M.: A bayesian networks approach for predicting protein-protein interactions from genomic data. Science **302**(5644), 449–453 (2003). doi:10.1126/science.1087361. http://www.sciencemag.org/content/302/5644/449.full.pdf
16. Chapple, C.E., Robisson, B., Spinelli, L., Guien, C., Becker, E., Brun, C.: Extreme multifunctional proteins identified from a human protein interaction network. Nature communications **6** (2015)
17. Albert, R.: Scale-free networks in cell biology. Journal of Cell Science **118**(21), 4947–4957 (2005). doi:10.1242/jcs.02714
18. Janjić, V., Sharan, R., Pržulj, N.: Modelling the yeast interactome. Scientific reports **4** (2014)
19. Vandin, F., Upfal, E., Raphael, B.J.: Algorithms for detecting significantly mutated pathways in cancer. Journal of Computational Biology **18**(3), 507–522 (2011)
20. Leiserson, M.D., Vandin, F., Wu, H.-T., Dobson, J.R., Eldridge, J.V., Thomas, J.L., Papoutsaki, A., Kim, Y., Niu, B., McLellan, M., *et al.*: Pan-cancer network analysis identifies combinations of rare somatic mutations across pathways and protein complexes. Nature genetics **47**(2), 106–114 (2015)
21. Batagelj, V., Zaversnik, M.: An O(m) algorithm for cores decomposition of networks. CoRR **cs.DS/0310049** (2003)
22. Chiba, N., Nishizeki, T.: Arboricity and subgraph listing algorithms. SIAM J. Comput. **14**(1), 210–223 (1985). doi:10.1137/0214017
